# United States’ Nursing Home Finances: Spending, Profitability, and Capital Structure

**DOI:** 10.1177/27551938231221509

**Published:** 2023-12-19

**Authors:** Charlene Harrington, Richard Mollot, Robert Tyler Braun, Dunc Williams

**Affiliations:** 1 Department of Social & Behavioral Sciences, 2345 University of California, San Francisco, CA, USA; 2 Long Term Care Community Coalition, New York, NY, USA; 3 12295 Weill Cornell Medical College, Cornell University, New York, NY, USA; 4 Department of Health Care Leadership and Management, College of Health Professions, Medical University of South Carolina, Charleston, SC, USA

**Keywords:** transparency, profits, related-parties, expenditures, revenues

## Abstract

Little is known about nursing home (NH) financial status in the United States even though most NH care is publicly funded. To address this gap, this descriptive study used 2019 Medicare cost reports to examine NH revenues, expenditures, net income, related-party expenses, expense categories, and capital structure. After a cleaning process for all free-standing NHs, a study population of 11,752 NHs was examined. NHs had total net revenues of US$126 billion and a profit of US$730 million (0.58%) in 2019. When US$6.4 billion in disallowed costs and US$3.9 billion in non-cash depreciation expenses were excluded, the profit margin was 8.84 percent. About 77 percent of NHs reported US$11 billion in payments to related-party organizations (9.54% of net revenues). Overall spending for direct care was 66 percent of net revenues, including 27 percent on nursing, in contrast to 34 percent spent on administration, capital, other, and profits. Finally, NHs had long-term debts that outweighed their total available financing. The study shows the value of analyzing cost reports. It indicates the need to ensure greater accuracy and completeness of cost reports, financial transparency, and accountability for government funding, with implications for policy changes to improve rate setting and spending limits.

Studies over many years have documented poor quality care and inadequate staffing in nursing homes (NHs) in the United States.^1–4^ While the public focus is generally on care and conditions of long-term residents, who are typically among the oldest and most frail individuals, even short-stay Medicare NH residents have a high rate of adverse events and deaths,^5^ and high rates of hospital readmissions for common and preventable problems.^6^ In light of these longstanding and widespread problems, the National Academics of Science, Engineering and Medicine recently issued a report calling for immediate action to initiate fundamental changes to improve the quality and lives of nursing home residents.^7^

Poor NH quality has been associated with low nurse staffing levels, particularly low registered nurse (RN) staffing.^7,8^ In 2017–2018, about 75 percent of NHs almost never met the Centers for Medicare & Medicaid Services (CMS) expected RN staffing levels based on resident acuity.^9^ Other studies show that most NHs failed to meet the CMS 2001 recommended minimum staffing levels in 2019 (4.1 total nurse staffing hours per resident day, including 0.75 RN hours per resident day^10^) and staffing levels recommended by experts based on acuity in 2017.^11,12^ In addition to putting residents at risk, heavy workloads, low wages and benefits, and poor working conditions have been associated with persistent staff dissatisfaction, shortages, and high staff turnover levels.^7,13,14^

NHs are primarily funded by the government (59%^15^), including Medicare, which pays for short-term, rehabilitation care, and Medicaid (funded jointly by the state and federal governments) which pays for long-term care.^16^ Medicare's prospective payment system is adjusted for the estimated staffing needs to meet facility-reported resident acuity, while giving NHs wide discretion in spending.^17,18^ State Medicaid reimbursement methodologies and rates vary widely; some states conduct audits and have imposed requirements for financial accountability.^19^

In 2019, the Medicare Payment Advisory Commission (MedPAC) reported that the Medicare fee-for-service programs spent about $27.8 billion (all dollar amounts in U.S. dollars) on free-standing NH services for about 1.5 million Medicare beneficiaries in 15,000 NHs.^16^ In addition, Medicaid spending was about $39 billion.^16^ While MedPAC reported that the total NH profit margin for all payers including Medicaid was 0.6 percent,^16^ the 2019 profit margin on Medicare spending was 11.3 percent and has remained over 10 percent for the past 20 years. Medicare rates for post-acute care are higher than Medicare managed care rates and Medicaid rates for long-stay residents. MedPAC has long reported that Medicare is overpaying for NH services.^16^

The American Health Care Association, the country's largest association of NH providers, has long claimed that Medicaid reimbursements cover only 70–80 percent of NH costs and that more than half of NHs operate at a loss.^20^ The Medicaid and CHIP Payment and Access Commission, however, reported that median 2019 state Medicaid facility-allowed base rates were 86 percent of reported facility costs, with some states providing supplemental rates.^21^ Medicaid residents tend to be long-stay residents with less intensive needs than Medicare post-acute residents.^7^ NHs with very high Medicaid resident days generally have lower staffing levels.^22^ A recent study found that California NHs had increased profits between 2019 and 2020 and that the percentage of Medicaid resident days were not associated with lower profits until Medicaid resident days were higher than 70 percent of total days.^23^

The American Health Care Association has also claimed that the low Medicaid reimbursement has put many NHs at financial risk of closing.^24^ There are, however, many reasons for NH closures, including low occupancy rates associated with the increasing percentage of Medicaid dollars spent on home and community-based services, which is now greater than institutional spending.^25^ Increased enrollment in Medicare and Medicaid managed care organizations (that have financial incentives to reduce nursing home use) have also played a role in the decreasing numbers of U.S. NHs.^26^ To a far lesser extent, actions taken by government regulators against individual facilities for poor quality have also resulted in NH closures.^27^ The fragmented payment system and payment policies have been the subject of debate in terms of how to improve NH care and financial transparency.^7^

## Methodology

### Specific Aims

Although the MedPAC reports overall NH spending annually, little is known about the financial status of NHs.^7^ This study was designed to fill in gaps about NH financial status and transparency to inform federal and state policy makers. Using the 2019 Medicare cost reports, this descriptive study examined the spending, profitability, and capital structure of U.S. NHs. The first aim was to examine NH revenues, expenses, profits and losses. To understand profits and losses, we excluded disallowances and depreciation. Disallowances are expenses that are not allowed under government payment policies, such as excess payments beyond the fair market value for goods and services and, therefore, should not be included as expenses when calculating profit margins. Depreciation is a non-cash NH expense that varies in accounting procedures and use across organizations. It is an estimated reduction in value of a fixed asset to account for the wearing out, consumption, or other loss arising from use over time or market changes. We excluded depreciation because it is an estimated non-cash expense and because we wanted to standardize expenses across NHs.

The second aim was to examine related-party expenditures. Related parties are separate corporate structures with the same or related owners, such as for property, management, nursing, and other companies.^28^ The third aim was to categorize expenses by comparing nursing, ancillary, support services, and benefits to expenditures on capital, administration, other costs, and profits. The final aim was to examine NH capital structure: assets, liabilities, and fund balances.

### Study Population and Data Source

The study population included all free-standing, nongovernment NHs in the United States reporting Medicare NH cost report data to CMS for 2019. Hospital NHs were excluded because their data are reported in hospital cost reports.

Because NHs report to Medicare based on each organization's fiscal year, the study examined 14,471 NH cost reports for the fiscal year that ended between July 1, 2019, through June 30, 2020. The study excluded 1,595 NHs that reported fewer than 360 days in a cost report period, 97 NHs with more than 370 days in a cost report period, 2 NHs that were in U.S. territories, and 1,041 facilities that were government-owned. After aligning the NH fiscal year dates with calendar years in our cleaning process, another 16 facilities were included. The final study population was 11,752 NHs.

### Study Design and Measures

This study examined selected measures from the Medicare cost reports. For details on cost report requirements, see the CMS Medicare provider reimbursement manual for NHs.^29^

#### Facility utilization and payer mix

We reported on the total number of beds, the total Medicaid, Medicare, and other payer days of care and total inpatient days, and the average occupancy rate. These characteristics show the size of facilities, the payment mix, and number of residents.

#### Revenues, expenses, net income, disallowances, and depreciation

Net revenues represent the actual income facilities received from inpatient services and other income sources, such as outpatient services. Net revenues from inpatient services were reported by subtracting contractual allowances and discounts from gross inpatient revenues. Total other income from other sources including outpatient services was combined with net inpatient revenues to report total net revenues for facilities.

Operating expenses were the costs of providing services. Total operating expenses were reported, which included disallowed expenses and depreciation.

Disallowances refer to specific costs or charges that are not permitted under Medicare reimbursement guidelines.^29^ In the context of NHs, examining disallowances is crucial as they directly impact the facility's total profit and loss margins. For example, when certain costs are not disallowed, NH allocated expenses are increased, which leads to a lower total income margin. Conversely, excluding disallowances from our analysis presents a more favorable picture of the total margins by omitting these nonreimbursable costs.

NHs are required to report Medicare disallowed expenses identified by CMS as: (1) those needed to adjust expenses to reflect actual expenses incurred; (2) those items which constitute recovery of expenses through sales, charges, fees, grants, gifts; (3) those items needed to adjust expenses in accordance with Medicare principles of reimbursement; and (4) those items which are provided for separately in the cost apportionment process.^29^ The disallowances include any payment for goods and services that exceed the fair market value for services. Specially, the instruction manual for the worksheet on disallowances states: “In accordance with 42 CFR 413.9(c)(3), if your operating costs include amounts not related to patient care (specifically not reimbursable under the program) or amounts flowing from the provision of luxury items or services (i.e., those items or services substantially in excess of or more expensive than those generally considered necessary for the provision of needed health services), such amounts are not allowable.”^29^ Disallowances include non-care-related expenses such as lobby association dues and legal fees.

Depreciation is a non-cash allowable expense used to allocate the cost of tangible or physical assets over their useful life. Depreciation accounting processes are estimated values that vary across facilities (within generally accepted accounting principles established by the Financial Standards Accounting Board). We removed depreciation to allow for a consistent or standardized profit measure.

Net income is the total profit or loss for a facility in dollars, while the total margin is the profit or loss expressed as a percentage of revenues. The net income from inpatient services (inpatient revenue minus net operating expenses) was reported. The net income or loss from all inpatient and outpatient services was reported (net income minus total expenses). Total margin was the net income or loss divided by net revenues.

The total income or loss from all services excluding disallowances was reported (by removing disallowed expenses from net revenues). Total margin excluding disallowed expenses was reported (the total income or loss excluding disallowances divided by net revenues). We also showed the total income and total margin after excluding disallowances and depreciation.

#### Related-party transactions

Most NHs are part of an enterprise with a myriad of related-party entities connected by ownership and control, such as real estate holding companies, management companies, pharmacies, and medical supply companies.^28^ Therefore, we reported the total payments by NHs to related-party companies.

Regulation 42 CFR 413.17(a) states that “costs applicable to services, facilities, and supplies furnished to the provider by organizations related to the provider by common ownership or control are includable in the allowable cost of the provider at the cost to the related organization.”^29^ As such, NHs are required to remove related-party profits through adjustments to operating expenses. The Medicare Cost Report instructions state, “the rationale behind this policy is that when you are dealing with a related organization, you are essentially dealing with yourself. Therefore, your costs are considered equal to the cost of the related organization”.^29^ The instruction manual explains that the allowable cost may not exceed the fair market price. These requirements apply to capital costs, supplies, and other costs paid to related parties.

In this study, 27 percent of NHs reported a credit to their related-party disallowed expenses which may indicate that related parties could have charged more for goods and services under the fair market rules than the payments they actually received. We examined these credits separately.

#### Net expenditures by cost categories

We wanted to assess how NHs spent their revenues; therefore we developed seven general cost categories to examine.

1. Inpatient Nursing Services: included all routine nursing service costs.
2. Ancillary Services: included: radiology, laboratory, all therapy services, electro-cardiology, medical supplies and drugs charged to patients, dental care, and other services.
3. Support Services: included plant operations, maintenance and repairs, laundry and linen service, housekeeping, dietary, nursing administration, central services and supply, pharmacy, medical records and library, social services, nursing and allied health education, and other general service costs.
4. Employee Benefits: costs for benefits for employees in all categories.
5. Administrative Costs: general administrative expenses, which include management fees, licensing and other regulatory fees, marketing expenses, bad debt, travel expenses, legal fees and settlements, and malpractice and insurance costs.
6. Capital Costs: included the costs for land, buildings and fixtures, and moveable equipment. These costs included leases and rentals for facilities and/or equipment, accrued interest related to land or depreciable assets used for patient care, property insurance, property taxes, and depreciation on assets used for patient care.^29^
7. Other Costs: included outpatient services, other reimbursable and special purpose costs.

#### Capital structure: assets, liabilities, fund balances, and financial ratios

In financial accounting, an asset is any resource owned or controlled by an organization that has a positive economic value. Assets represent value to owners that can be converted into cash. Total assets were reported for NHs including: (1) current assets such as cash on hand and in banks, accounts receivable and other receivables; (2) fixed assets such as land, buildings, and equipment; (3) other assets such as investments, leases, and funds due to and from owners and officers.^29^

Assets are funded through a mix of debt (liabilities) and equity (fund balances). Total liabilities are the debts or financial obligations of a facility. These included: (1) current liabilities, including accounts payable, salaries, wages and fees payable, and other payables, and (2) long term liabilities, including mortgages, notes, loans, loans from owners, and other liabilities.^29^

The fund balance (or equity) represents the total accumulation of operating surpluses and deficits (profits and losses) after cash distribution to owners. This is commonly described as the ownership interest or retained earnings by for-profit entities or net assets by nonprofit entities.

From the assets, liabilities and fund balance, we examined two capital structure measures. First, the debt-to-capital ratio measures the financial leverage of a company. This was calculated by comparing its total liabilities to total capital (assets), or the proportion of debt that a business uses to fund its ongoing operations as compared with capital. Second, the debt-to-equity ratio was calculated by dividing liabilities (both short- and long-term) by the fund balance. The total debt-to-equity ratio is a solvency measure that shows the proportion of debt a company uses to finance its assets, relative to the amount of equity financing. A higher ratio indicates a company is more highly leveraged (in debt), which carries a higher risk of insolvency.

### Analysis

Each NH had only one unique cost report for 2019. For each measure, we made assumptions related to whether missing values were truly missing or should be coded as zeros. For example, we coded any missing values for services or items that all NHs may not have as a zero. Alternatively, if a NH reported nothing for revenues, we coded that as missing since it was not possible for an operating NH to have no revenues. Generally, variables without dollar values were assumed to be zero.

Because some NH-reported data had outliers, we winsorized the distribution by limiting the outliers to the top and bottom one percent for the revenues, expenses, and other variables in the study. This was done to limit the effect of outliers or abnormal extreme values on the calculations. We did not use the winsorization process when calculating ratios because the original data had already been winsorized. We also tested and excluded ratios we considered to be impossible (e.g., greater than 100% Medicare payer mix) or improbable (e.g., when the absolute value of the net margin was greater than 60%).

We calculated the net inpatient revenue as well as total net revenues (including inpatient and outpatient and other revenues). Expenses, disallowances, depreciation, and related-party transactions were calculated as described. The percentage of each expenditure category was calculated relative to adjusted operating expenses, net inpatient revenues, and total net revenues.

## Results

### Facility Utilization and Payer Mix

As noted, the study examined 11,752 NHs in 2019 with over 1.35 million licensed beds (mean of 115; standard deviation [SD] of 59) and over 400 million days of care (mean 34,108; SD 18,835) in 2019 (see Table 1). Of the total days of care, 53 percent were days paid by Medicaid, 11 percent were days paid by Medicare, and 36 percent were days paid by other payers. Average occupancy was 81 percent.

**Table 1.** U.S. Nursing Home Utilization and Payer Mix, Revenues, Expenses, and Net Income Margin, 2019.

	Medicare cost report reference	Mean	S.D.	Total
*Facility utilization and payer mix*				
Number of facilities				11,752
Number of beds	S-3, Col 1, Line 8	115	59	1,355,309
Total Medicaid days	S-3, Col 5, Line 8	18,184	13,544	213,699,471
Total Medicare days	S-3, Col 4, Line 8	3,767	3,303	44,267,319
Total other days	S-3, Col 3 plus Col 6	12,158	13,885	142,872,368
Total inpatient days	S-3, Col 7, Line 8	34,108	18,835	400,839,158
Average occupancy rate				81.03%
*Revenues*				
Net inpatient revenues	G-3, Line 3	$ 10,020,635	$ 6,591,251	$ 117,702,380,393
Total other income	G-3. Line 25	$ 727,722	$ 2,592,511	$ 8,552,187,841
Total net revenues	G-3, Line 3 plus Line 25	$ 10,748,729	$ 7,467,940	$ 126,254,568,234
*Expenses*				
Total operating expenses	G-3, Line 4	$ 10,692,678	$ 7,394,659	$ 125,660,354,113
Disallowed expenses	A-8, Line 100, Col 2	$ (546,183)	$ 735,163	$ (6,418,813,477)
Allowable expenses	G-3, Line 4 minus A-8, Line 100, Col 2	$ 10,147,834	$ 7,035,739	$ 119,237,045,030
Depreciation	S-2 Part 1, Line 23, Column 2	$ 337,813	$ 732,912	$ 3,969,975,556
Allowable expenses without depreciation	G-3, Line 4 minus A-8, Line 100, Col 2	$ 9,812,068	$ 6,604,950	$ 115,252,546,400
*Net Income*				
Net income from inpatient services	G-3, Line 5	$ (627,305)	$ 2,585,083	$ (7,372,085,255)
Net income from all services	G-3, Line 31	$ 62,205	$ 1,201,683	$ 730,102,193
Net margin	G-3, Line 31/G-3, Line 3 + Line 25			0.58%
Net income excluding disallowances	G-3, Line 31 minus A-8, Line 100, Col 2	$ 598,696	$ 1,862,254	$ 7,148,915,670
Net margin excluding disallowances	G-3, Line 31 minus A-8, Line 100, Col 2/G-3, Line 3, +Line 25			5.66%
Net income excluding disallowances and depreciation	G-3, Line 31 + A-8, Line 100, Col 2 + S-2 Part 1, Line 23, Column 2	$ 936,502	$ 2,023,545	$ 11,118,891,226
Net margin excluding disallowances and depreciation	G-3, Line 31 + A-8, Line 100, Col 2 + S-2 Part 1, Line 23, Column 2/ G-3, Line 3 plus Line 25			8.84%

### Revenues, Expenses, Net Income, Disallowances, and Depreciation

The NH net inpatient revenues totaled $117.70 billion (mean $10,020,635; SD $6,591,251) and other income was $8.55 billion (mean $727,722; SD $2,592,511) for the year. Overall, total net revenues were $126.25 billion (mean $10,748,729; SD $7,467,940) in 2019 (see Table 1).

Total operating expenses were $125.66 billion (mean $10,692,678; S.D. $7,394,659). Total Medicare disallowed expenses (including related-party disallowances) were $6.42 billion (mean $−546,183; SD $735,163; or 5.11% of total expenses) in 2019, resulting in total allowable expenses of $119.24 billion (see Table 1). Disallowed expenditures were reported by 99 percent of NHs. Total depreciation (non-cash) expense was $3.97 billion (mean $337,813; SD $732,912), or 3.16 percent of expenses. Overall, total allowable expenses minus the non-cash depreciation charges were $115 billion (mean $9,812,068; SD $6,604,950).

Net income from inpatient services, including disallowed expenses, showed a loss of $7.37 billion (mean −$627,305; SD $2,585,083) in 2019 (see Table 1). Net income from all services was $730 million (mean $62,205; SD $1,201,683) and the total margin from all services was 0.58 percent, which ranged from a net gain of 56 percent to a net loss of 131 percent. In respect to total net income (including disallowances), 45 percent of all NHs reported a net loss.

**Figure 1.**
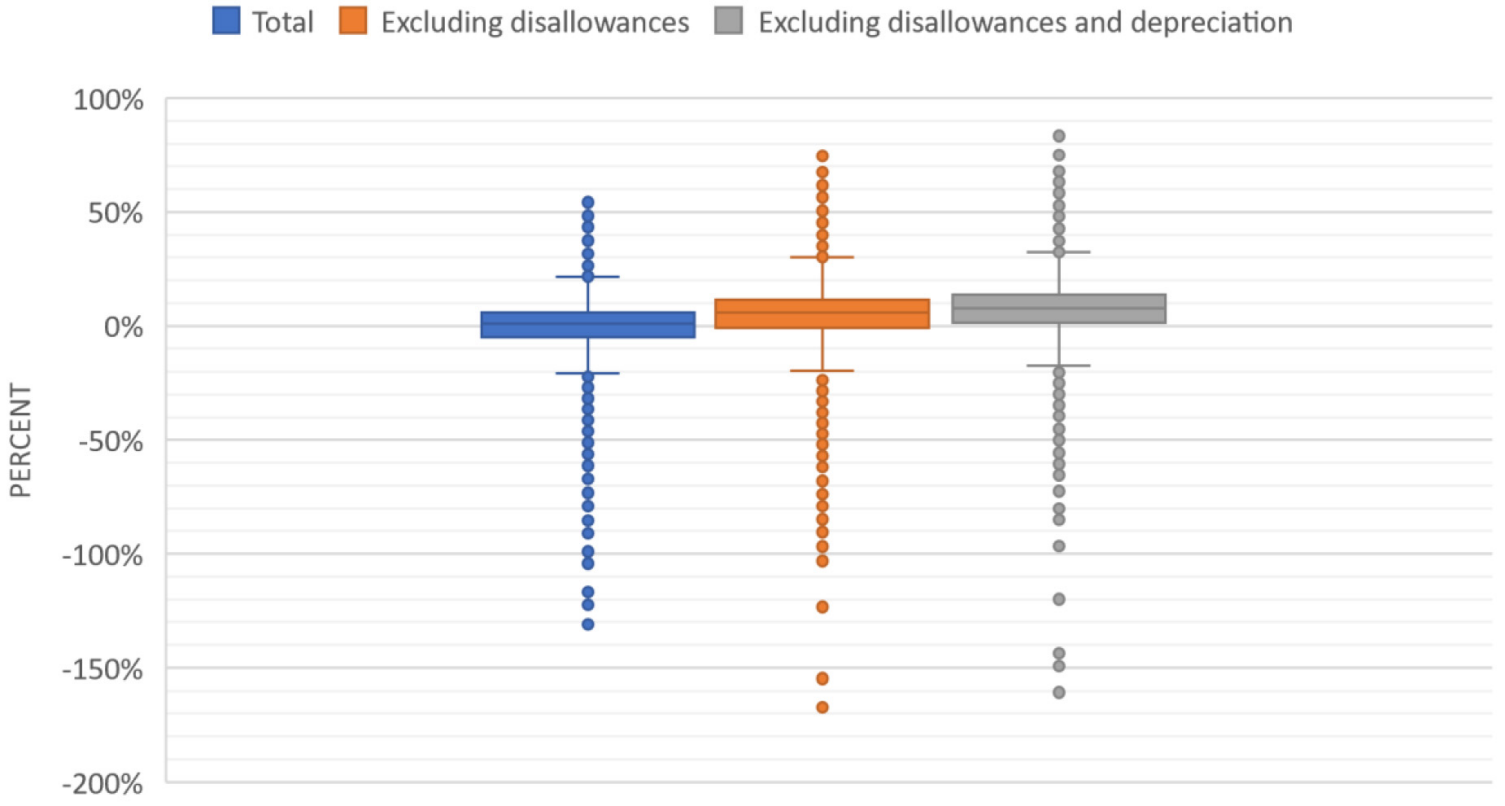
Net income margin.

Net income excluding disallowances was $7.15 billion (mean $598,696; SD $1,862,254), with a net margin of 5.66 percent, which ranged from a net gain of 77 percent to a net loss of 167 percent (after the winsorization process, which limited the outliers to the top and bottom 1%). When disallowances and depreciation were excluded, the total income was $11.12 billion and the total margin was 8.84 percent (ranging from a profit of 83% to loss of 161%). When disallowances and depreciation were excluded from net income, 79 percent of NHs reported a profit. Figure 1 shows the distribution of NH net income margins before and after disallowances and depreciation expenses were removed (after winsorization).

### Related-parties Expenditures

Table 2 shows that 9,001 NHs (76.6%) reported making $11.23 billion in payments to related parties in 2019. Of the facilities reporting related-party costs, 5,788 NHs (64.30%) reported $1.95 billion in related-party payments that were disallowed. In addition, 3,213 NHs (35.70%) reported the fair market value for comparable goods and services was higher than the payments the related parties received from the NH. Therefore, these NHs reported a credit to their total disallowed payments of $764.76 million to reduce disallowed payments. Overall, NHs reported that 9.54 percent of their total net operating revenues were paid to related-party organizations.

**Table 2.** U.S. Nursing Home Related-Party Expenditures, 2019.

Related-Party Payments					
Payments to related parties	A-8-1, Line 10, Col 5	$ 955,539	$ 1,049,365	$ 11,229,488,526	N = 9,001 (76.6%)
Disallowed related-party payments	A-8-1, Line 10, Col 6	$ (337,412)	$ 395,939	$ (1,952,943,209)	N = 5,788 (64.30%)
Credits to disallowed payments	A-8-1, Line 10, Col 6	$ 238,022	$ 288,064	$ 764,763,961	N = 3,213 (35.70%)
No related-party payments				0	N = 2,751 (23.4%)
% of related-party payments compared to total net operating revenue	A-8-1, Line 10, Col 5 / G-3 Line 3			9.54%	

### Net Expenditures

Table 3 shows the adjusted net expenditures for selected categories of expenses after removing disallowances and depreciation. We compared the net expenditures to operating expenses, net inpatient revenues, and total net revenues. Inpatient services and nursing were $34.41 billion (27.25% of total net revenues of $126.25 billion). Ancillary services were $12.16 billion (9.63% of total net revenues). Support services totaled $26.99 billion (21.38% of total net revenues) and employee benefits added to $9.43 billion (7.47% of total net revenues). Administrative expenses were $17.91 billion (14.19%), capital expenses were $12.56 billion (9.95%), and other expenses were $1.64 billion (1.30%). All direct service-related costs amounted to 65.73 percent of total net revenues, while administration, capital, other costs, and profits were 34.27 percent of total net revenues.

**Table 3.** U.S. Nursing Home Net Expenditures and Net Margins by Payment Category, 2019.

Net Expenditures After Adjustments (Excludes Disallowances and Depreciation)				
	Medicare Cost Report	Mean	S.D.	Total
Inpatient services and nursing	A, Lines 30 to 33, Col 7	$ 2,927,590	$1,816,131	$ 34,405,041,728
Ancillary services	A, Lines 40 to 52, Col 7	$ 1,034,934	$ 712,419	$ 12,162,543,969
Support services	A, Lines 5 to 15, Col 7	$ 2,296,709	$1,658,266	$ 26,990,929,727
Employee benefits	A, Line 3, Col 7	$ 802,359	$ 809,992	$ 9,429,323,684
Administrative expenses	A, Line 4, Col 7	$ 1,524,086	$1,002,789	$ 17,911,057,622
Capital expenses	A, Line 1 + 2, Col 7	$ 1,068,438	$1,304,558	$ 12,556,280,225
Other expenses and non-reimbursable costs	A, Lines 60 to 63, 70–74, 80–84, 90–95, Col 7	$ –	$ –	$ 1,636,488,716
Total costs	A, Line 100, Col 7	$ 9,654,116	$7,304,155	$ 115,091,665,671

Percent of Net Expenditures		% of Adjusted Operating Expenses	% of Net Inpatient Revenues	% of Total Net Revenues
Inpatient services and nursing	A, Lines 5 to 15, Col 7	29.89%	29.23%	27.25%
Ancillary services	A, Lines 30 to 33, Col 7	10.57%	10.33%	9.63%
Support services	A, Lines 5 to 15, Col 7	23.45%	22.93%	21.38%
Employee benefits	A, Line 3, Col 7	8.19%	8.01%	7.47%
Administrative expenses	A, Line 4, Col 7	15.56%	15.22%	14.19%
Capital expenses	A, Line 1 plus 2 Col 7	10.91%	10.67%	9.95%
Other expenses and non-reimbursable costs	A, Lines 60 to 63, 70–74, 80–84, 90–95, Col 7	1.42%	1.39%	1.30%
Subtotal		100%	97.78%	91.16%
Total net margin^a^			2.22%	8.84%
Total			100.00%	100.00%

Source: CMS Medicare Nursing Home Cost Reports for 2019; https://data.cms.gov/provider-compliance/cost-report/skilled-nursing-facility-cost-report/data.

^a^ Difference between net expenditures (without disallowances and depreciation) and the net revenues.

### Capital Structure: Assets, Liabilities, Fund Balance and Financial Ratios

Table 4 shows that total NH assets were $154.48 billion (mean $13 million; S.D. $30.9 million) in 2019, which included about $10.28 billion in cash on hand and in banks. Of the total NHs, 4.06 percent reported no assets or negative assets. Because many nursing homes are owned by chains, it is likely the cash for these facilities is maintained at a system level. Thus, accounting practices may result in subsidiary facilities not reporting any cash on hand.

**Table 4.** U.S. Nursing Home Capital Structure: Assets, Liabilities, Fund Balance, and Financial Ratios, 2019.

Assets, Liabilities, and Fund Balance					
Account	Medicare Cost Report Reference	Mean	S.D.	Total	Number
Total Assets	G, Line 34, Column 1–4	$ 13,100,000	$ 30,900,000	$154,481,713,378	11,752
Cash on hand and in banks	G Line 1, Column 1–4	$ 874,857	$ 2,442,672	$ 10,281,316,322	
Total Liabilities	G, Line 51, Column 1–4	$ 11,500,000	$ 32,400,000	$135,383,761,992	
Total Fund Balance	G, Line 59, Column 1–4	$ 1,600,000	$ 606,000	$ 19,089,286,797	
Debt to capitalization ratio	Liabilities/ Total assets	52.45%	28.07%		N = 6,889
Debt to equity ratio	Liabilities/ Fund balance	3.20	6.07		N = 7,073

Liabilities were reported to be $135.38 billion (mean $11.5 million; S.D. $32.4 million). In 2019, 525 facilities (4.55%) out of 11,752 NHs reported either zero or negative liabilities.

NHs reported a fund balance of $19.09 billion (mean $1,600,000; SD $606,000). Of the total NHs, the average debt-to-capital ratio was 52.45 percent (SD 28.07), and the debt-to-equity ratio was 3.20 percent (SD 6.07).

## Discussion

This study found that U.S. NHs had net inpatient revenues of $117.70 billion and total net revenues of $126.25 billion (average of $10.75 million per NH) in 2019. NHs reported a loss on inpatient operations of $7.37 billion but a net profit of $730 million on their operations. The NH net profit margin for all payers including Medicaid was 0.58 percent in 2019, which was the same amount reported by MedPAC for 2019.^16^

When disallowed costs (including related-party disallowances) were excluded from expenditures, the total margin was 5.66 percent. Further, when the disallowances and depreciation expenses were excluded, the 2019 total margin was 8.84 percent. This total margin showed a wide range of profits (up to 83% profit) and losses (up to 161%), after the winsorization process adjusted for outliers.

The disallowed costs of $6.42 billion, including related-party disallowances, were 5.08 percent of total net revenues. As noted, the Medicare disallowed expenses were for expenses not related to patient care. This would include association dues, legal fees, and related-party disallowances that exceed the fair market value of comparable goods and services in the marketplace. It should be noted, however, that not all disallowed expenses are profits. For example, 44 states had mandatory state provider taxes for nursing facilities in 2022 (to increase their federal Medicaid matching funds), but the amount of these state provider taxes was not reported.^30^

The Medicare prospective payment system neither requires NHs to return disallowed costs nor CMS to make facility-specific rate adjustments for the coming year that take into account prior year disallowed costs.^31^ Because disallowed expenses are not required to be paid back to Medicare and the NH has discretion over these expenditures, they should be excluded to more accurately reflect NH income margins. Because CMS does not require facilities to return disallowances, overall Medicare reimbursement rates are inflated. This policy appears to result in compounding the inflation of rates on an annual basis. It is worth noting that, in respect to Medicaid, some states may adjust for disallowances. For example, the California Medicaid program conducts audits and makes adjustments for disallowances in its facility-specific rates for the coming year but does not require disallowed expenses to be returned to Medicaid.^23,32^

NHs can legally do business with related-party organizations. Approximately 77 percent of NHs in this study reported $11 billion (9.54% of revenues) in related-party payments in 2019. NHs reported $1.95 billion in disallowed related-party expenses because payments exceeded the fair market value of comparable goods and services in the marketplace. The Medicare cost reports do not require NHs to submit detailed reports on related-party service expenses, profits and losses, or administrative costs that can be used to verify the disallowed costs. Therefore, some profits and administrative costs may not be identified, and some related-party organizations may not be identified.^33^ The accuracy of the self-reported related-party disallowances cannot be determined without an audit.

In 2023, the National Academics of Science, Engineering and Medicine Committee concluded that NHs may be using related-party and unrelated party entities to hide profits. They identified the underlying problems of inadequate financial transparency and inaccurate and incomplete cost reporting and urged policy changes to improve transparency and accountability.^7^ One study of California NHs found that NHs with related-party transactions were more likely to report lower profit margins,^23^ suggesting that the use of related-party organizations may be a successful way to hide profits.

Related-party transactions are facing increased scrutiny by state and federal regulators to determine if federal regulations are followed and if funds are diverted away from resident care.^34–36^ For example, the New York State Attorney General recently filed a lawsuit against four NHs alleging for multiple fraudulent schemes to divert government funds through related-party real estate arrangements, unnecessary and exorbitant loans with inflated interest rates, phony fees paid to companies they and their family members own, and paying themselves inflated salaries for work that was not performed. The lawsuit alleges that the diversion of funds led to shortages of staffing and significant resident neglect, harm, and humiliation.^37^ The findings from our study suggest the need for more transparency of related-party transactions.

This study examined how NHs spend their revenues. NH net expenses, without disallowed costs and depreciation, were 27 percent on inpatient services and nursing, 10 percent on ancillary services, 21 percent on support services, and seven percent on employee benefits in 2019. Of total net revenues, about 66 percent was spent on direct care services.

Administrative expenses were 14 percent, capital expenses were approximately 10 percent, and other expenses one percent for a total of 25 percent of total net revenues, and the total margin was reported to be about nine percent profit, after excluding disallowances and depreciation.

Expenditures were about 66 percent on direct services compared to 34 percent for administration, capital, and profits. This finding suggests that NHs may be able to shift some spending from administration, capital, and profits toward staffing, wages and benefits, and resident services. Medical loss ratio legislation, adopted in the Patient Protection and Affordable Care Act, requires health insurance companies to spend a certain proportion of premiums on medical claims and activities rather than on administration and other profits.^38^ If the government required a certain percentage of total NH revenues to be spent on direct care, this could improve both financial accountability and quality of care.

Four states (New Jersey, New York, Massachusetts, and Pennsylvania) have passed legislation requiring a percentage of NH revenues to be spent on direct care services, with limitations on administrative costs, property costs, and profits.^39^ For example, New York's legislation requires at least 70 percent of total operating expenses for direct care, including 40 percent for resident-facing staffing, and limited profits to no more than 5 percent of expenses. These states require NHs to return funds that exceed the state limits.^39^

In terms of the NH capital structure, the study found that the average debt-to-capital ratio was 3.20 percent. Ratios of >1 show that debt exceeds equity. NHs also reported a debt-to-equity ratio (52.45%). There are many reasons for NHs to take on debt such as to finance expansion and improvement, deduct debt from taxable income to reduce tax burden, maintain control over an organization rather than bringing in other owners or investors, or earn more on investments than the cost of the debt. Too much debt, however, is associated with a higher risk of insolvency.

Private equity (PE)-owned NHs have been criticized for loading companies with excessive debt, selling off assets and real estate of acquired companies, taking advantage of tax loopholes, and charging high management fees.^40,41^ Such financial schemes may have consequences for NH quality and costs. A recent national cohort study found that long-stay residents of PE firm–owned nursing homes were more likely to have an ambulatory care-sensitive emergency department visit and hospitalization after acquisition compared with residents of non-PE firm–owned, for-profit nursing homes. The PE firm-owned nursing homes also had higher total Medicare costs.^42^

A few NHs reported small or no assets or debts. One possible reason might be the segregation of assets and liabilities, where the operational aspect of a NH (i.e., care provision, staffing, etc.) is kept separate from the real estate and property aspect, which may be owned by a related-party or a different entity altogether. This arrangement can serve to protect the property assets from operational liabilities and lawsuits.^28^

Studies have reported that many NHs have placed their land and buildings in separate property companies that are related-party or un-related organizations.^28,43,44^ Other NHs have sold or placed property into real estate investment trusts (REITs), which may or may not be related parties. One recent study reported that REITs owned 12 percent of NHs and had $116.8 billion in NH assets in 2019.^45^

Additionally, some NHs might have paid off any initial startup or acquisition loans a long time ago, and they may currently operate without needing to incur additional debts. However, the lack of reported debts can also be indicative of financial strategies to optimize balance sheets for various reasons, such as attracting investors or for tax benefits. It is also plausible that certain reporting nuances or accounting practices allow for the deferment or restructuring of debt, making it appear as low in certain reports. While the cost reports do not provide a direct linkage between NHs reporting few debts and their affiliations with separate property entities or REITs, it is important to consider how such structures might serve protective, operational, or financial optimization purposes.

In terms of limitations, we used Medicare NH cost reports, which have been shown to have inaccuracies.^46^ Medicare cost reports, however, are the only publicly available source of information about NH financial revenues and spending nationally.^47^ Although this study implemented extensive cleaning procedures to improve accuracy, there may be missing and inaccurate data. Another potential limitation is that, due to NH-specific fiscal year reporting periods, some cost reports covered March through June of 2020 at the beginning of the COVID-19 pandemic, which could have impacted revenues and expenditures during that period.

A U.S. General Accountability Office (GAO) study of NH cost report data from 2011–2014 found that CMS does little to ensure the accuracy and completeness of the data.^47^ The GAO recommended that CMS should make data accessible to the public and ensure data reliability because of the importance of this data source. Despite these recommendations in 2016, CMS NH cost reports do not have computerized accuracy checks for simple math errors, and cost reports are not required to be audited or validated by an accounting professional before they are submitted to CMS. In addition, the cost report data only show the amount of cash at the end of the year and not the total cash removed by NH owners throughout the year. As noted above, the cost reports do not require sufficient information to verify disallowances, especially on the profits and administrative costs on related-party transactions. Because CMS claims to use NH cost report data for determining its Medicare rates annually, there is a clear need to improve the accuracy and completeness of the reports.^48^

Medicare may conduct financial audits in situations where fraud may be suspected or to confirm provider charges for bad debts resulting from Medicare deductible and coinsurance amounts which are uncollectible from Medicare beneficiaries. As a general rule, however, CMS does not conduct Medicare financial audits on NHs. CMS has no requirements for consolidated cost reports that provide information on corporate chains or entire companies that own NHs. Moreover, CMS has no penalties for false reporting, even though this could improve the accuracy and transparency of cost reports.^41^

In conclusion, this study provided an in-depth report on U.S. NH financial status for 2019. When disallowances and depreciation were excluded, total income margins increased from less than one percent to approximately nine percent. Most NHs (77%) reported related-party transactions that accounted for about 10 percent of net revenues, suggesting these transactions require further study as to whether they are accurately reported and whether funds are diverted away from needed resident care. Disallowances, including those from related parties, are not required to be returned to Medicare, thereby increasing NH income margins. Finally, approximately 66 percent of net expenses were for direct care compared to 34 percent for administration, capital, and profits. Policy changes may improve the proportional spending on direct care and patient quality.

Overall, the study findings illustrate the value of analyzing NH financial performance using cost reports. They also suggest that there are potential policy options that could be implemented to improve rate setting, reimbursement methods, the accuracy and the transparency of cost reports, including related-party transactions, and the accountability for spending government funds.
